# Functional Properties of the MAP Kinase UeKpp2 in *Ustilago esculenta*

**DOI:** 10.3389/fmicb.2020.01053

**Published:** 2020-06-09

**Authors:** Yafen Zhang, Yingli Hu, Qianchao Cao, Yumei Yin, Wenqiang Xia, Haifeng Cui, Xiaoping Yu, Zihong Ye

**Affiliations:** Zhejiang Provincial Key Laboratory of Biometrology and Inspection & Quarantine, College of Life Sciences, China Jiliang University, Hangzhou, China

**Keywords:** *Ustilago esculenta*, UeKpp2, mating, filamentous growth, UeRbf1, UeUkc1

## Abstract

*Ustilago esculenta* undergoes an endophytic life cycle in *Zizania latifolia*. It induces the stem of its host to swell, forming the edible galls called *jiaobai* in China, which are the second most commonly cultivated aquatic vegetable in China. *Z. latifolia* raised for *jiaobai* can only reproduce asexually because the *U. esculenta* infection completely inhibits flowering. The infection and proliferation in the host plants during the formation of edible gall differ from those of conventional pathogens. Previous studies have shown a close relationship between mitogen-activated protein kinase (MAPK) and fungal pathogenesis. In this study, we explored the functional properties of the MAPK UeKpp2. Cross-species complementation assays were carried out, which indicated a functional complementation between the *UeKpp2* of *U*. *esculenta* and the *Kpp2* of *Ustilago maydis*. Next, *UeKpp2* mutants of the UeT14 and the UeT55 sporidia background were generated; these showed an aberrant morphology of budding cells, and attenuated mating and filamentous growth *in vitro*, in the context of normal pathogenicity. Interestingly, we identified another protein kinase, UeUkc1, which acted downstream of UeKpp2 and may participate in the regulation of cell shape. We also found a defect of filamentous growth in *UeKpp2* mutants that was not related to a defect of the induction of mating-type genes but was directly related to a defect in *UeRbf1* induction. Overall, our results indicate an important role for UeKpp2 in *U*. *esculenta* that is slightly different from those reported for other smut fungi.

## Introduction

The smut fungus *Ustilago esculenta* induces a swollen stem in *Zizania latifolia*, its only known host (Chung and Tzeng, 2004). *Z. latifolia* individuals colonized with *U*. *esculenta* are widely cultivated in East and Southeast Asia and are the second most commonly cultivated aquatic vegetable grown in China, where they are called *jiaobai* because their swollen stems are delicious and nutritious (Suzuki et al., 2012; Jiang et al., 2016; Jose et al., 2016; Yan et al., 2018). Due to the fungal infection, *jiaobai* cannot flower or produce seeds (Guo et al., 2015). It reproduces asexually. It is now generally accepted that there are two type strains of *U*. *esculenta*, T and MT. The T type strain shows the pathogenic life cycle of typical smut fungi, with three distinct stages (Zhang et al., 2017): the budding growth stage of haploid cells from diploid teliospore germination; the mating stage, which is a prerequisite for infection; and pathogenicity development, with proliferation and teliospore formation happening *in planta*. The mating stage includes two steps: fusion of two compatible haploid cells, mainly regulated by a pheromone receptor system that consists of pheromones and pheromone receptors (encoded at the triallelic *a* mating-type locus), and post-fusion filamentation that is capable of infection, controlled by the active heterodimeric bE/bW complex (Liang et al., 2019; Zhang et al., 2019). The T type strain causes gray *jiaobai*, which is discarded by farmers due to its unacceptable taste and potential to trigger hypersensitivity pneumonitis (Fujii et al., 2007). However, there are also galls with a white appearance, called white *jiaobai* in China. These are valued by farmers, and contain an inner tissue full of fungal hyphae, probably due to colonization by the MT type strain (Ye et al., 2017). It is widely believed that in white *jiaobai*, *U*. *esculenta* only grows within the stems during plant development and overwinters in the rhizomes that are left for reproduction (Zhang et al., 2012; Jose et al., 2016). Studies have found that the MT type strain, which shows an endophytic life in the host, has defects at every stage of its typical life cycle, showing a multi-budding morphology, a slower growth rate in haploid cells, delayed conjugation tube formation and confined hyphal growth during mating, and an attenuated ability to proliferate and form teliospores (Zhang et al., 2017). However, knowledge of the molecular basis for the pathogenic development of *U*. *esculenta* is very limited.

Pathogenic development in smut fungi is closely related to mating. Its mating system consists of both *a* and *b* mating-type loci (Bakkeren et al., 2008; Raudaskoski and Kothe, 2010; Zuo et al., 2019). The recognition of pheromones by opposite pheromone receptors, encoded at the *a* locus, elicits the fusion of two compatible haploid cells (Szabo et al., 2002; Zhang et al., 2019). The heterodimeric transcription factor bE/bW, which is encoded by the *b* locus, triggers filamentation and pathogenicity (Yan et al., 2016; Zhang et al., 2019). The cross-talk between the highly conserved cAMP pathway and the mitogen-activated protein kinase (MAPK) pathway is crucial for mating by integrating pheromone signaling and environmental cues (Feldbrügge et al., 2006).

In eukaryotic cells, the MAPK signaling pathways are involved in the regulation of developmental processes through the transduction of extracellular signals (Hamel et al., 2012; Jiang et al., 2018). Five MAPK pathways have been identified in *Saccharomyces cerevisiae*, among which the Fus3/Kss1 MAPK pathway, an evolutionarily conserved MAPK module, is responsible for mating, filamentous growth, and invasive growth (Zhao et al., 2007). In fungi, Fus3/Kss1 homologs are conserved in an activation loop (the A-loop), including the TXY dual phosphorylation site, which is phosphorylated by upstream MAPKK and is essential for kinase activity (Chen et al., 2001). However, the mechanism of regulation of Fus3/Kss1 homologs is not conserved in fungi, due to the different needs relating to different environments.

In *S. cerevisiae*, Kss1 responds to starvation signaling by regulating filamentous growth through Ste12, which is tethered by Tec1 to TCS elements upstream of filamentation genes (Chou et al., 2006). Fus3, responding to a pheromone, activates Far1, a bifunctional protein required for polarization and G(1) arrest to repress G(1)-S specific transcription (Breitkreutz et al., 2001). Together with Kss1, it also activates Ste12, a transcription factor that triggers mating processes by regulating mating genes through the Ste12 binding site (Breitkreutz et al., 2001). Additionally, Fus3 can be autophosphorylated by allosteric Ste5, resulting in downregulation of transcriptional output responding to pheromone signaling, ensuring a tuned quantitative pathway through its input–output property (Bhattacharyya et al., 2006).

In *Ustilago maydis*, a homolog of the yeast Fus3/Kss1 MAPK pathway has been identified, consisting of MAPKKK Kpp4, MAPKK Fuz7, and MAPK Kpp2/Kpp6, responding to pheromone signaling or plant surface signals to regulate the formation of filamentous dikaryons and fungal virulence (Vollmeister et al., 2012). It directly regulates Prf1, both at the transcriptional and the post-transcriptional level, through promoter discrimination phosphorylation; this activates a defined pheromone-responsive linear transcriptional cascade bE/bW > Rbf1, which is essential for filamentous growth and further pathogenic development (Kaffarnik et al., 2003; Zarnack et al., 2008; Vollmeister et al., 2012). Rbf1 may also be directly induced by Prf1, which is regulated by activated Kpp2 (Zarnack et al., 2008). However, this does not seem important, and the details of the regulation mechanism are not known. In addition, Kpp2 is involved in the regulation of pheromone-induced cell cycle arrest in the G2 phase and the formation of conjugation tubes, independently of Prf1 (Garcia-Muse et al., 2003).

There are also reports of Fus3/Kss1 homologs in other pathogenic fungi. In *Sporisorium scitamineum*, SsKpp2 is required for mating and filamentation. This occurs through the integrated regulation of the conserved pheromone signal transduction pathway and fungal quorum-sensing (QS) signal (Deng et al., 2018). In *Tilletia indica*, TiKpp2 is induced by host factors in a time-dependent manner and participates in myelination growth and pathogenicity by activating the downstream transcription factor Prf1 (Gupta et al., 2013). In *Magnaporthe oryzae*, Pmk1 is responsible for appressoria formation and cell-to-cell invasion by responding to plant cues (Zhao et al., 2005; Sakulkoo et al., 2018). In *Candida albicans*, Cek1 and Cek2 are functionally redundant in the dimorphic switch process, virulence, and cell wall integrity (Correia et al., 2016).

In *U*. *esculenta*, we identified UeFuz7 and UePrf1, which participate in mating and filamentation (Zhang et al., 2018b). In addition, we identified the Fus3/Kss1 homolog UeKpp2, which interacts with UeFuz7 and UePrf1 and is induced by mating and infection (Zhang et al., 2018a). In this study, we explored the functional properties of UeKpp2 in the life cycle of *U*. *esculenta*, including budding growth, stages of mating, and the development of pathogenicity.

## Materials and Methods

### Strains and Plant Growth Conditions

The *Escherichia coli* strain JM109 (Takara) was used for cloning purposes. The compatible haploid T type strains UeT14 (a1b1 CCTCC AF 2015016) and UeT55 (a2b2 CCTCC AF 2015015) and their derivatives (listed in Supplementary Table S1) were used in this study. The strains of *U*. *maydis* used in this study are also listed in Supplementary Table S1. The growth conditions and media for *E*. *coli* (Russell and Sambrook, 2001), *U*. *maydis* (Holliday, 1974), and *U*. *esculenta* (Zhang et al., 2019) have previously been described. The growth conditions of *Zea mays* (the early golden bantam) and the wild *Z*. *latifolia* used for pathogenic development assays of tested strains before and after stem injection have previously been described (Flor-Parra et al., 2007; Zhang et al., 2019).

### Plasmid and Strain Construction

In the deletion of genes in *U*. *esculenta*, a PCR-based approach using hygromycin as the resistance marker was used as previously described (Yu et al., 2015). With the UeT14 genomic DNA as template, the ∼1 kb long left-border and right-border fragments adjacent to the target gene were amplified by PCR, using the primer pairs gene-UF/UR and gene-DF/DR. Both primers have ∼25 bp specific homology arms of hygromycin resistance genes. The hygromycin resistance gene with its promoter was separated into two fragments (up and down), with a ∼450 bp overlap, using PCR with the primers Hyg-F/Hyg3 and Hyg4/Hyg-R, respectively. Then, the left (right) border fragments of the target gene were ligated to the 5′ end (the 3′ end) of the up (down) fragment of the hygromycin resistance gene via fusion PCR using the primer pairs gene-UF/Hyg3 (Hyg4/gene-DR). The two resulting fragments were transformed into protoplast cells of distinct *U*. *esculenta* strains to generate target gene deletion strains through homologous recombination, following a PEG/CaCl_2_-mediated protoplast transformation method (Yu et al., 2015). First, the primer pairs gene-verity-F/R for object gene detection, Hyg-verity-F/R for hygromycin resistance gene detection, and gene-F3/MF167 and MF168/gene-R3 for insertion site detection were used in the preliminary screening of transformants. qRT-PCR (with the primer pair gene-QF/R) and Southern hybridization (PCR-probe amplified with primers of Hyg-verity-F/R and gene-verity-F/R) were used for further confirmation.

For complementation of the *U*. *maydis* strain SG200Δkpp2, the *Kpp2* gene promoter sequence was cloned from genomic DNA of the haploid solopathogenic *U*. *maydis* strain SG200 with primers PF1/PR1 (or PF1/PR2). Then the cDNA of the *U*. *esculenta* strain UeT14 (or the *U*. *maydis* strain SG200) was used as template to amplify the open reading frame of *UeKpp2* (or *Kpp2*) using primers UeKpp2-CF/CR (or Kpp2-CF/CR). The plasmid P123 was linearized by *Hin*dIII and *Not*I to the 4.6 kb genomic region. The above three fragments were recombined using ClonExpress^®^ II MultiS One Step Cloning Kit (Vazyme, C113–01) and transformed into *E*. *coli* to obtain a plasmid, which was linearized by *Ssp*I and transformed into the *U*. *maydis* strain SG200Δkpp2 to generate the strains SG200Δkpp2:UeKpp2 and SG200Δkpp2:Kpp2. To complement the UeKpp2 deletion strain, *UeKpp2* open-reading frame was PCR amplified using the primer pairs UeKpp2-CF1/CR and cloned into plasmid pUMa932 between the *Nco*I and *Not*I sites using ClonExpress^®^ II MultiS One Step Cloning Kit (Vazyme, C113–01). Similarly, *UeUkc1* open-reading frame was PCR amplified and plasmid pUMa932-UeUkc1 was generated. The resulting two plasmids were linearized by *Nde*I and transformed into UeT14Δkpp2 and UeT55Δkpp2 to generate the strains UeT14ΔUeKpp2:UeKpp2, UeT55ΔUeKpp2:UeKpp2, UeT14△UeKpp2:UeUkc1, and UeT55△UeKpp2:UeUkc1. The transformants were selected using regeneration agar containing carboxin. The selected transformants were further confirmed based on gene expression levels.

To generate the strains UeTSPΔUeKpp2:P_b_UeRbf1, adopting the genomic DNA of UeT14 as a template, ∼1 kb left border and right border fragments adjacent to *UeKpp2* were amplified by PCR using the primer pairs UeKpp2-UF/UR and UeKpp2-DF1/DR. These primers have ∼20 bp specific homology arms of the 5′ end of the hygromycin resistance genes and of the 3′ end of the *UeRbf1* gene. Additionally, the promoter of the *bW2* gene, the open reading frame of the *UeRbf1* gene, and the hygromycin resistance genes were PCR amplified using primer pairs bW2-PF/PR, UeRbf1-CF/CR, and Hyg-F/Hyg-R. Then these were cloned and ligated by fusion PCR. Next, the fusion fragment was separated into two fragments (up and down) with a ∼450 bp overlap, using PCR amplification and the primers Hyg-F/Hyg3 and Hyg4/Hyg-R, respectively. Finally, the up fragment was ligated to the left border fragments of *UeKpp2*, and the down fragment was ligated to the right border fragments of *UeKpp2*, using fusion PCR with the primer pairs UeKpp2-UF/Hyg3 (Hyg4/UeKpp2-DR). As with the gene-deletion process, the two constructed fragments were transformed into *U*. *esculenta* protoplast and analyzed via PCR/RT-PCR/Southern blot to confirm the replacement of UeKpp2 by UeRbf1 in the UeTSP strain, its single insertion, and its expression levels.

All primers used above are listed in Supplementary Table S2.

### Mating Assays

The mating assays of *U*. *esculenta* were performed following Zhang et al. (2017). Haploid isolates were collected by centrifugation after liquid expansion of the culture and adjusted to an OD_600_ of ∼2.0. Then equal amounts of compatible test strains were mixed. Next, 2 μL drops of this mixture were cultured on YEPS solid medium (2 μL *U*. *maydis* SG200 and its derivatives were spotted on PDA solid medium) and cultured at 28°C for 60 h observation at 12 h intervals.

### Plant Infection Assays

For *U*. *esculenta* inoculation assays, 20-day-old seedlings of *Z*. *latifolia* were used. Following Zhang et al. (2017), compatible strains with an OD_600_ of ∼2.0 were mixed at a 1:1 ratio and syringe-inoculated into seedlings, which then were cultured in a greenhouse under a 12/12 h light/dark cycle at 25 ± 2°C and 70% relative humidity. For the infection of maize seedlings, SG200 and its derivatives were cultured and resuspended in water to an OD_600_ of ∼2.0; then, samples were syringe-inoculated into 7-day-old maize seedlings, following Gillissen et al. (1992).

### Light Microscopy and Confocal Microscopy

For microscope observation of cell morphology, we used an inverted microscope (Nikon Ti-S inverted microscope, NT-88-V3 micro-operating system). For colony morphology observation, we used a stereo microscope (Nikon stereo microscope). A confocal microscope (Leica Microsystems) was used to examine fungal colonization of the leaf sheath. Fungal hyphae were stained with wheat germ agglutinin–Alexa Fluor 488 (WGA, Sigma, L4895). Samples from infected plants were destained with ethanol and placed into 10% KOH at 85°C for 3 h, washed twice with PBS (140 mM NaCl, 16 mM Na_2_HPO_4_, 2 mM KH_2_PO_4_, and 3.75 mM KCl, pH 7.5), and vacuum-infiltrated with PBS containing 10 μg mL^–1^ WGA for 20 min at intervals of 10 min, following Doehlemann et al. (2008). WGA Fluor 488 was excited at 488 nm, and emitted fluorescence was detected in the 495–530 nm range. The images were processed using LAS-AF software (Leica Microsystems).

### Real-Time PCR

Real-time PCR was conducted to detect gene expression. Samples of distinct strains from the budding growth stage, the mating stage, and the infection stage were collected at the selected relevant times. CFX Connect^TM^ Real-Time System (Bio-Rad, United States) was used in combination with Platinum SYBR Green qPCR Premix EX TaqTM (TliRNaseH Plus) (Takara, Japan) for detection, and iCycler software (Bio-Rad) was used for data analyses. β*-Actin* was used as the internal reference for measuring gene expression. The experiment had three biological and three technical replicates. Relative expression was determined using the 2^–ΔCt^ method, with values of *p* < 0.05 considered significant. All primers are listed in Supplementary Table S2.

## Results

### Functional Complementation Between UeKpp2 of *U*. *esculenta* and Kpp2 of *U*. *maydis*

**FIGURE 1:**
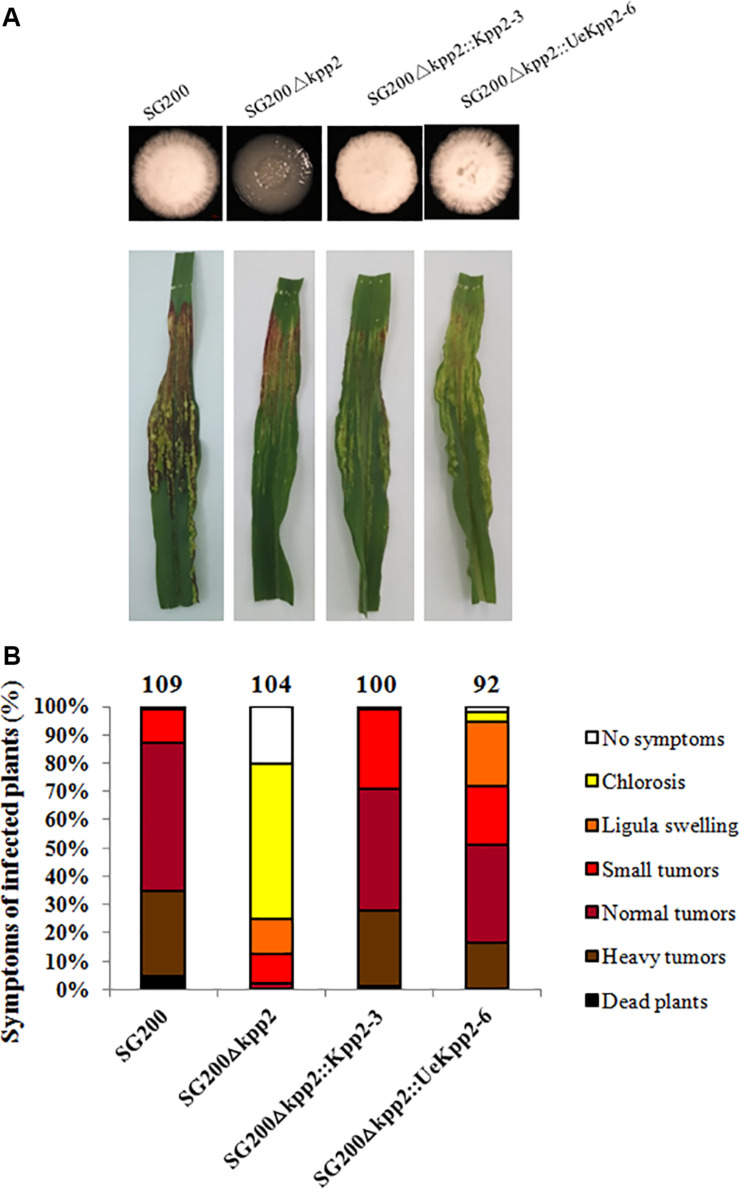
UeKpp2 is capable of complementing the SG200Δkpp2 mutant phenotype. **(A)** Solopathogenic strains derived from SG200 were tested for filamentous growth on PDA plates. Images were taken after incubation for 72 h at 28°C. Filamentous growth appeared white and fuzzy. **(B)** The disease symptoms and ratings of the solopathogenic strains derived from SG200 are indicated below each column. The strains used to inoculate the 7-day-old corn seedlings are indicated below each column. The disease symptoms were scored 12 days after infection. Photographs of the typical symptoms are given in the top row. The symptoms were grouped into one of seven categories according to the most severe symptoms displayed in a given plant, following Kämper et al. (2006). The groups are color-coded in order of severity on the right of the diagram. The mean values of the three independent infections are indicated above the respective columns, as are the total number of infected plants.

UeKpp2 in *U*. *esculenta* has an amino acid identity of 96% to Kpp2 in *U*. *maydis*, and the TEY dual phosphorylation sites are conserved (Zhang et al., 2018a). Cross-species complementation assays were carried out to explore the homologies of UeKpp2 to Kpp2 in terms of function. The coding sequences for *UeKpp2* and *Kpp2* were introduced into the *U*. *maydis* SG200Δkpp2 strain (Müller et al., 2000), under the native promoter of *Kpp2* to exclude problems with promoter strength or time of expression. The derived single-copy strains SG200Δkpp2:Kpp2-3 and SG200Δkpp2:UeKpp2-6 were selected after being verified by Southern blot analyses (Supplementary Figure S1). All of the strains, including SG200 and SG200Δkpp2, were cultured on PDA plates and subjected to a virulence assay. Filamentous growth was inhibited (Figure 1A) and a few tumors were formed (Figure 1B) in SG200Δkpp2, consistent with previous results that mutation of *Kpp2* reduces pathogenic development (Müller et al., 2000). However, the SG200Δkpp2:UeKpp2-6 and SG200Δkpp2:Kpp2-3 strains appeared as fuzzy colonies *in vitro* (Figure 1A) and showed severe disease phenotypes after inoculation, comparable to those of SG200 (Figure 1B). These results illustrate that UeKpp2 is capable of complementing the SG200Δkpp2 mutant phenotype, indicating the potential role of UeKpp2 in mating and pathogenicity.

### Deletion of *UeKpp2* in *U*. *esculenta* Alters Budding Cell Morphology

We separately knocked out *UeKpp2* in wild-type strains UeT14 and UeT55, which were selected after validation by Southern blot and qRT-PCR analyses to ensure a single and correct insertion (Supplementary Figure S2). The mutant strains, when grown on a solid YEPS medium, showed no differences in growth rate from WT strains (Supplementary Figure S3). Nitrogen starvation (BM medium) and stressful conditions, including cell wall stress (0.5 mM Congo red), hyperosmotic stress (500 mM NaCl), and oxidative stress (1 mM H_2_O_2_) (Deng et al., 2018), slowed cell growth in both the WT strains and *UeKpp2* mutants. However, these growth rates did not differ between WT and mutant strains (Supplementary Figure S3).

**FIGURE 2:**
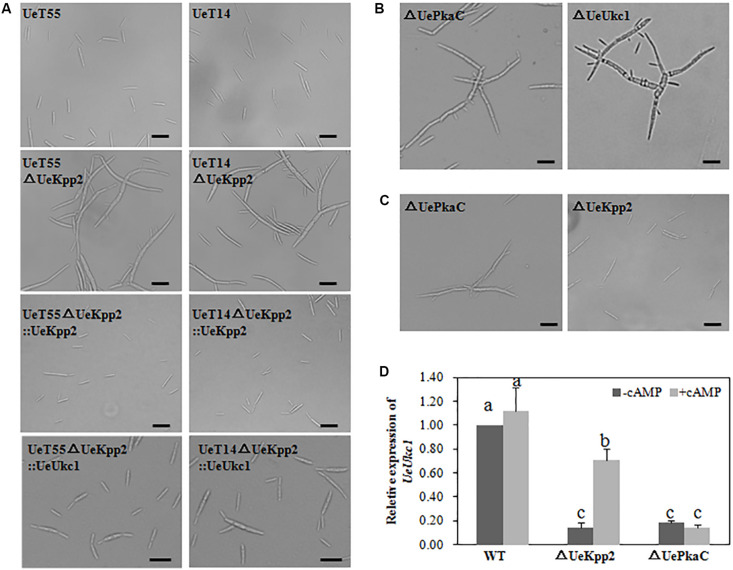
UeKpp2 is involved in the regulation of the morphology of budding cells but not in stress response. **(A)** Microscopic imaging of budding cells of the WT strains, *UeKpp2* mutants, *UeKpp2* reconstituted strains or *UeUkc1* over-expressed strain in *UeKpp2* mutants, 24 h after spotted culture on YEPS medium. Scale bar = 20 μm. **(B)** Microscopic imaging of budding cells of *UePkaC* mutant or *UeUkc1* mutant in UeT14 strain, 24 h after spotted culture on YEPS medium. The scale bar = 20 μm. **(C)** Microscopic imaging of budding cells of *UePkaC* mutant or *UeKpp2* mutant in UeT14 strain, 24 h after spotted culture on YEPS medium supplemented with 20 mM cAMP. Scale bar = 20 μm. **(D)** Relative expression of *UeUkc1* in WT, *UePkaC* mutant or *UeKpp2* mutant, 24 h after spotted culture on YEPS medium (–cAMP) or YEPS medium supplemented with 20 mM cAMP (+cAMP). The letters above the columns indicated significant differences at *p* < 0.05 (Tukey). Each strain was cultured in liquid YEPS medium to an OD_600_ of ∼0.8 and collected by centrifugation to reach the target concentration of 10^7^ cells per mL.

It is worth noting that the cell morphology of *UeKpp2* mutants changed. Compared to the short (19.4 ± 5.2 μm) and yeast-like budding cells of WT strains (Figure 2A), mutant cells were longer (28.9 ± 9.5 μm). In addition, most mutants formed long chains, in which cells were attached to each other or appeared elongated with multiple buds (Figure 2A). Moreover, this aberrant morphology was apparent under normal as well as stressed conditions (data not shown). To ensure that all potential mutant phenotypes were associated with this mutation, we also created reconstituted *UeKpp2* strains by restoring the ORF of *UeKpp2* into the *UeKpp2* mutant strain using the constitutive promoter Otef; the results were confirmed using qRT-PCR to ensure transcript restoration (Supplementary Figure S4). Microscopy observation showed that the reconstituted *UeKpp2* strains recovered the normal phenotype (Figure 2A), indicating a possible role of UeKpp2 in regulating the shape of yeast-like cells in budding growth.

Further, we found that a mutation of *UeUkc1* (accession number: MN845072) or *UePkaC* (accession number: ALM02104.1), genes we had identified in other studies, caused similar cell shapes to the *UeKpp2* mutants (Figure 2B and Supplementary Figure S2). Amino acid sequence analyses showed that UeUkc1 may be homologous to the nuclear Dbf2-related (NDR) kinase with a role in determining cell shape (Durrenberger and Kronstad, 1999), and that UePkaC may be homologous to the catalytic subunit of the protein kinase A in the cAMP-PKA signaling pathway (Supplementary Figure S5). Considering that cAMP-PKA signaling is involved in polar growth, which maintains normal cell morphology (Gold et al., 1994; Durrenberger et al., 1998) and cross-talks with MAPK signaling in many cases (Martinez-Espinoza et al., 2004; Meng and Zhang, 2013), we first analyzed the microscopic morphology of *UeKpp2* under cAMP treatment. *UeKpp2* mutants recovered normal cell shape in the YEPS medium when supplied with 20 mM cAMP, while the *UePkaC* mutants did not (Figure 2C). Also, because downregulation of *ukc1* results in a prolonged G2 phase, which leads to a change in cell shape (Sartorel and Perez-Martin, 2012), we assessed *UeUkc1* expression in *UeKpp2* and *UePkaC* mutants cultured for 24 h in YEPS medium or YEPS medium with 20 mM cAMP. There was an extremely significant reduction of *UeUkc1* in the mutants compared to WT strains. After cAMP treatment, the expression of *UeUkc1* was significantly upregulated in the *UeKpp2* mutants while there were no obvious expression changes in *UePkaC* mutants (Figure 2D). Furthermore, the cell shape of *UeKpp2* mutants recovered, whereas that of *UePkaC* mutants did not (Figure 2D), indicating that effective expression of *UeUkc1* is essential to maintain normal cell morphology. In addition, we improved the expression level of *UeUkc1* in the *UeKpp2* mutant by over-expressing *UeUkc1* under the constitutive promoter Otef (Supplementary Figure S4). The constructed strain showed a normal phenotype (Figure 2A). These findings indicated that UeKpp2 might regulate transcriptional induction of *UeUkc1* in cell shape regulation.

### Deletion of *UeKpp2* in *U*. *esculenta* Impairs Mating and Filamentous Growth *in vitro*

**FIGURE 3:**
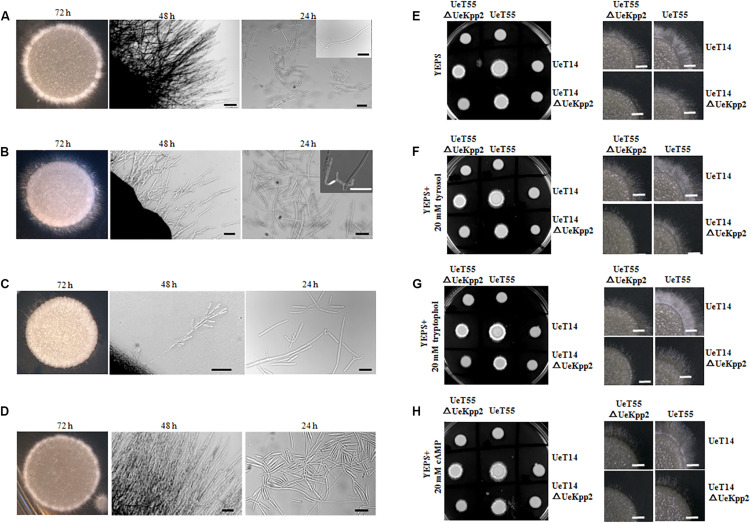
Deletion of *UeKpp2* impaired mating and filamentous growth. The morphology of colonies, hyphae, or cell fusion in the compatible WT strains UeT14 and UeT55 cross **(A)**, UeT14ΔUeKpp2 and UeT55 cross **(B)**, UeT14ΔUeKpp2 and UeT55ΔUeKpp2 cross **(C)**, or *UeKpp2* reconstituted strain cross **(D)**, separately. They were spotted onto YEPS medium. Photographs were taken at 72 h under inverted microscope and at 48 and 24 h under a stereo microscope. The scale bar represents 100 μm in 48 h and 20 μm in 24 h. The impact of QSMs and cAMP on mating and filamentous growth of WT and *Uekpp2* mutant was assessed in **(E)** YEPS solid medium, **(F)** YEPS supplemented with 20 mM tyrosol, **(G)** YEPS supplemented with 20 mM tryptophol, or **(H)** YEPS supplemented with 20 mM cAMP. The images on the left are colonies images in a culture dish taken by a camera at 96 h. The images on the right show the clearer hyphae growth status at 72 h by stereomicroscopy.

The mating and filamentous growth of *UeKpp2* mutants were assessed *in vitro* by co-spotting compatible combinations on YEPS plates. The compatible WT strains UeT14 and UeT55 with opposite mating types served as a positive control, where a white fuzzy appearance at the edge of the colony indicated successful mating and the formation of filaments (Figure 3A). However, filamentous growth was obviously inhibited in the compatible combination of *UeKpp2* mutants (Figure 3C), and recovered to a comparable level of control in the reconstituted strains (Figure 3D). The crosses between UeT14ΔUeKpp2 and UeT55 or between UeT14 and UeT55ΔUeKpp2 showed a delayed filamentous growth (Figure 3B). Further follow-up observations showed that normal fusion and filamentous growth appeared ∼24 h after culturing in WT strains (Figure 3A), while rare fusion cells appeared in the double mutant cross (Figure 3C) or in the cross between UeT14ΔUeKpp2 and UeT55 (Figure 3B). We also observed unusually long and branched conjugation tubes or branched and curly filaments in the cross of UeT14ΔUeKpp2 and UeT55 (Figure 3B) but relatively little formation of conjugation tubes in the double mutant cross, although cell fusion was not affected (Figure 3C). These findings indicate a defect in conjugation tube formation in *UeKpp2* mutants. Moreover, the radial filamentous growth almost disappeared in the double mutant cross and was impaired in the UeT14ΔUeKpp2 and UeT55 cross. Instead, hyphal tips were branched (Figures 3B,C). This phenomenon suggests that UeKpp2 also plays a role in filamentous growth.

In addition, we tested the effects of the cAMP-PKA signaling or fungal QSM signaling compounds tryptophol and tyrosol (Chen and Fink, 2006; Wongsuk et al., 2016) on the mating and filamentous growth of *UeKpp2* mutants (Figures 3E–H). The addition of tryptophol led to white aerial mycelium growing denser in the two compatible WT strains. However, it did not promote or restore mating or filamentation growth in *UeKpp2* mutants, where the defects of conjugation tube formation and radial filamentous growth appeared. On the other hand, cAMP, which restored the morphology of budding cells in *UeKpp2* mutant, had no impact on the mating/filamentous growth of this mutant.

### Delayed Formation of Conjugation Tubes in *UeKpp2* Mutants Is Not Related to a Gene Induction

**FIGURE 4:**
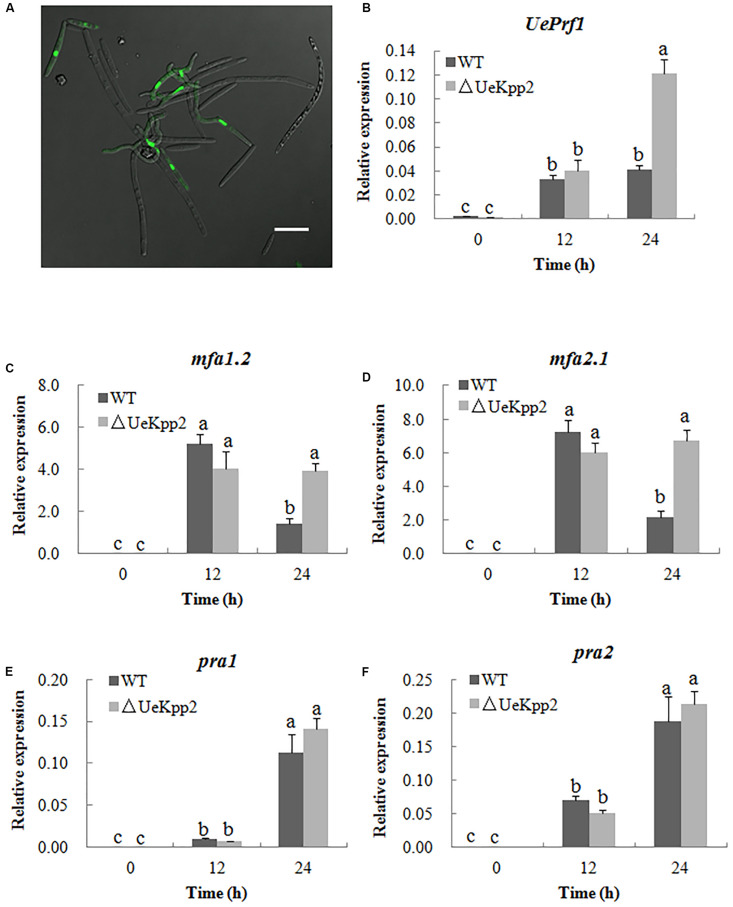
Deletion of *UeKpp2* delayed conjugation tubes formation but not impact the *a* genes and *UePrf1* induction. **(A)** Confocal microscopy of the cells 18 h after mating of UeT14ΔUeKpp2 and UeT55-EGFP strains cultured on YEPS medium. The conjugation tubes formed in the green fluorescent cells. Scale bar represents 20 μm. Relative expression of *UePrf1* **(B)**, *mfa1*.*2* **(C)**, *mfa2*.*1* **(D)**, *pra1* **(E)**, and *pra2* **(F)** in the wild-type strains UeT14 × UeT55 and the corresponding *UeKpp2* deletion strain during mating on YEPS medium. Samples were collected at 0, 12, and 24 h. The letters above the columns indicate significant differences at *p* < 0.05 (Tukey).

Earlier experiments have shown that the defect in the mating of *UeKpp2* mutant is mainly related to the formation of conjugation tubes. Furthermore, we introduced the *EGFP* over-expression strain UeT55-EGFP to examine the conjugation tubes in the crosses between UeT14ΔUeKpp2 and UeT55-EGFP, under a six-interval microscopic observation. Before 18 h culture, all of the conjugation tubes that were observed had formed in the green fluorescent cells (Figure 4A). This indicated that the delayed formation of the conjugation tubes only occurs in *UeKpp2* mutant cells.

As noted, the pheromone signaling pathway, including the *UePrf1* and *a* genes, is important for regulating conjugation formation (Zhang et al., 2018b). Thus, the expression levels of the *UePrf1* and *a* genes were checked in the double mutant cross compared to the WT strain cross, using qRT-PCR. Mutation of *UeKpp2* did not change the basic or induced expression levels of the genes tested at 12 h (Figures 4B–F). Interestingly, the *mfa* genes maintained high expression levels after being cultured for 24 h, although the levels decreased in controls (Figures 4C–F). Furthermore, the expression of *UePrf1* was induced to a higher level in mutants at 24 h than in WT strains (Figure 4B). These findings indicate that the delayed formation of conjugation tubes in *UeKpp2* mutants, which was not related to the defect in induction of *a* genes, has an unknown cause. It was also worth investigating the relationship between the prolonged induced expression of both *UePrf1* and *a* genes and the malformation of conjugation tubes and hyphae.

### Defective Filamentous Growth in *UeKpp2* Mutants Is Related to the Defect in *UeRbf1* Induction

**FIGURE 5:**
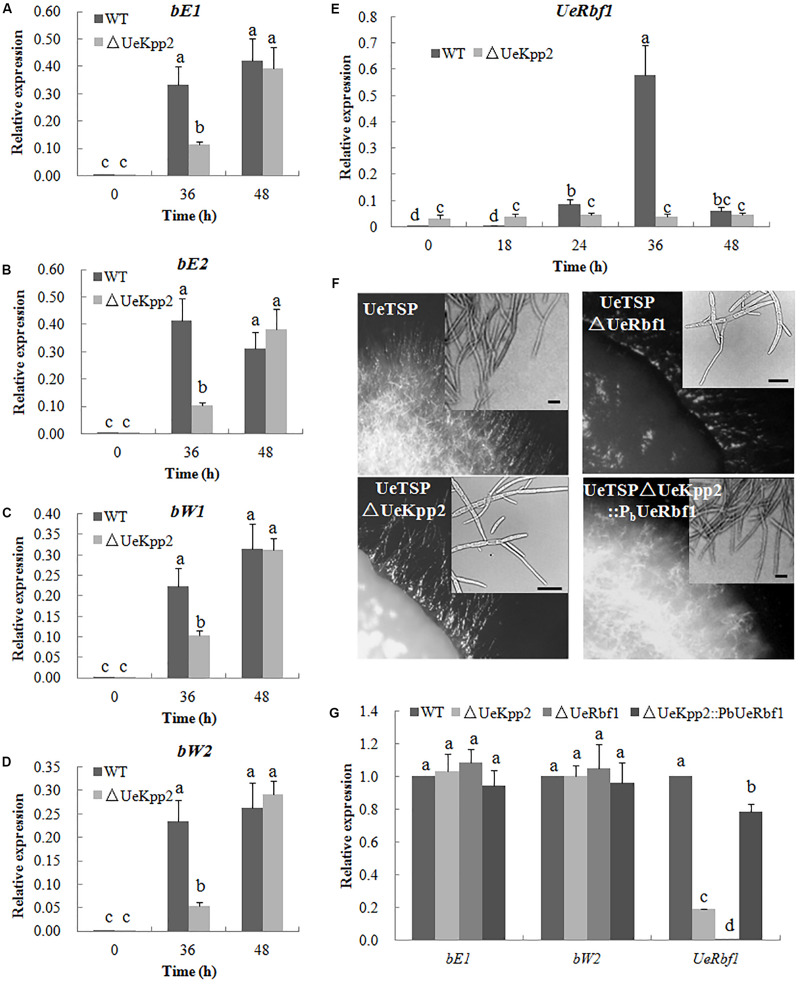
The defect of filamentous growth in *UeKpp2* mutants was related to the defect of *Rbf1* induction, but not the *b* genes induction. Relative expression of *bE1* **(A)**, *bE2* **(B)**, *bW1* **(C)**, *bW2* **(D)**, and *UeRbf1* **(E)** in WT and *UeKpp2* mutants during mating in YEPS medium. Samples were collected under a 12 h interval. Different letters above the columns indicate significant differences at *p* < 0.05 (Tukey). **(F)** Microscopy of the colonies and cells in solopathogenic strains UeTSP and their derived strains (UeTSPΔUeKpp2, UTSPΔUeRbf1, and UeTSPΔUeKpp2:P_b_UeRbf1). The panel in the up-right corner of the image of cell morphology shows the typical cells observed under inverted microscopy. The scale bar represents 20 μm. **(G)** Relative expression of *bE1*, *bW2*, and *UeRbf1* in UeTSP strain (CK), UTSPΔUeKpp2 strain, UTSPΔUeRbf1 strain and ΔUeKpp2:PbUeRbf1 strain. The letters above the columns indicate significant differences at *p* < 0.05 (Tukey).

Induced expression of either the active bE/bW heterodimer or Rbf1 triggers a dimorphic switch from budding yeast-like growth to filamentous tip growth in the typical smut fungi *U*. *maydis* (Heimel et al., 2010a, b). In *U*. *esculenta*, the *UeRbf1* and *b* genes also participate in filamentous growth (Hu et al., 2015; Zhang et al., 2019). First, we detected the expression levels of the *UeRbf1* and *b* genes in the *UeKpp2* double mutant cross relative to the WT strain cross during mating, using qRT-PCR. The results showed that the mutation of *UeKpp2* did not change the basic or induced expression levels of the genes when the expression levels were compared at 0 and 48 h. Additionally, at 36 h, the expression of *b* genes was obviously lower in the mutants than in WT strains, indicating delayed induction of *b* genes in mutants (Figures 5A–D). In the fusion process, the delayed induction of *b* genes may be simply related to delayed cell fusion. Further, the basic expression of *UeRbf1* is significantly higher in *UeKpp2* mutants than in WT strains, which appeared not to have significantly changed during mating. However, in WT strains during mating, *UeRbf1* was induced at 24 h, reached its highest level at 36 h, and then fell (Figure 5E). We also studied the UeTSP strain (a2b2:P_bE1_:bE1 P_mfa1.2_:mfa1.2) (Zhang et al., 2019), which showed filamentous growth and had a fuzzy appearance without mating. Consistent with the expected results, the deletion of *UeKpp2* and *UeRbf1* in the UeTSP strain showed similarly scarce filament formation (Figure 5F). Interestingly, the expression of *b* genes in mutants was comparable to that of the WT strains during the culture, but the expression of *UeRbf1* was markedly reduced in *UeKpp2* mutants, which showed a drastically reduced filamentation (Figure 5G). These results suggest that the mutated *UeKpp2* did not impact the expression of the *b* genes but did influence the induction of *UeRbf1*. To find support for this conjecture, a *UeRbf1* induction strain in the *UeKpp2* mutant was constructed under the influence of the promoter of *bW2* (UeTSPΔUeKpp2:PbUeRbf1). In this strain, *UeRbf1* was induced during the culture (Figure 5G). As expected, it recovered its filamentous growth and fuzzy appearance. All of these results suggest that the defective filamentous growth in *UeKpp2* mutants was related to the defect in *UeRbf1* induction, not the induction of *b* genes.

### UeKpp2 Is Not Required for Penetration, Proliferation, Teliospore Formation, or Germination in *U*. *esculenta*

**FIGURE 6:**
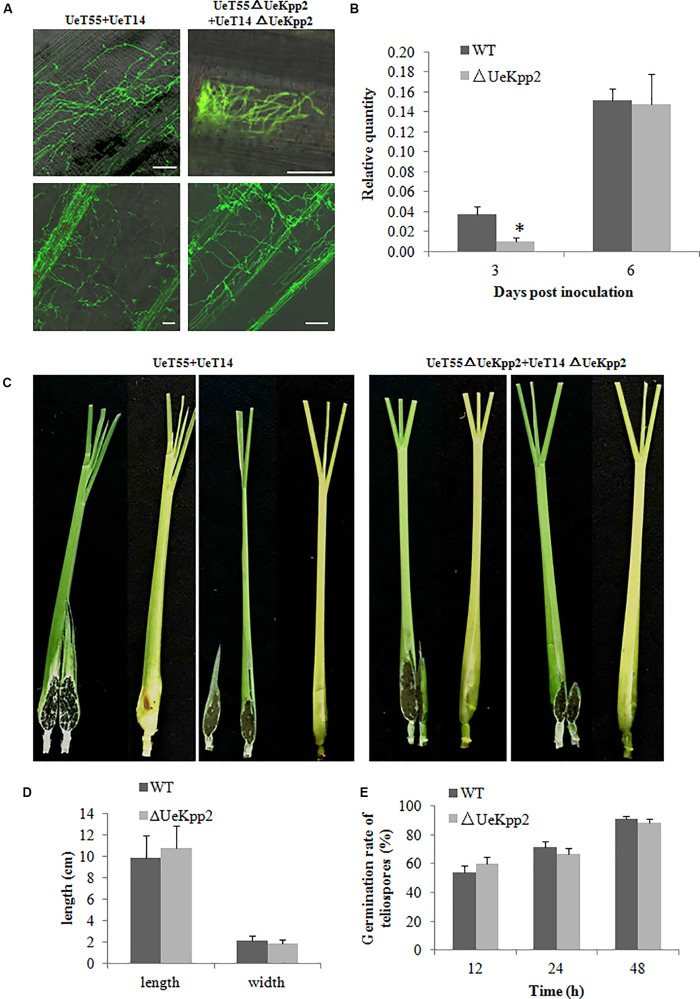
Deletion of *UeKpp2* has no influence on penetration, proliferation, teliospore formation, or germination in *U*. *esculenta*. **(A)** Observation of fungal infection status in leaf sheath. Samples infected with the wild-type strains UeT14 × UeT55 and the corresponding *UeKpp2* deletion strains were collected at 3 (above) and 6 dpi (below), stained with wheat germ agglutinin-Alexa Fluor 488 and analyzed using laser scanning confocal microscopy. The scale bar represents 50 μm. **(B)** Relative-quantity analyses of the DNA copies of *UeActin* and *ZlActin* to evaluate the hyphal amount. The samples were collected at 3 and 6 dpi. Asterisk denotes significant difference at *p* < 0.05. **(C)** Phenotypes of the swollen stems. Samples infected with the wild-type strains UeT14 × UeT55 (left) and the corresponding *UeKpp2* deletion strains (right) were collected at 90 dpi. Both formed swollen stems with full of teliospores. **(D)** The length and width of the swollen stems of the plants infected with the wild-type strains UeT14 × UeT55 and the corresponding *UeKpp2* deletion strains. **(E)** The germination rate of the teliospores of *UeKpp2* mutant and WT strains. An appropriate amount of teliospores were collected from plants after 90 days inoculation by *UeKpp2* mutant or WT strains, dispersed in water to get equal concentrations, and incubation on YEPS plates at 28°C. Light microscopy was taken after 12, 24, and 48 h culture. The numbers of germination teliospores were analyzed. There were no significant differences in the teliospores germination rate between *UeKpp2* mutant and WT strains.

An inoculation test was carried out to test the pathogenicity of the *UeKpp2* mutant. At 3 days post inoculation (dpi), there was infectious hyphal growth of WT strains (mixed with UeT14 and UeT55) but few infectious hyphae in the *UeKpp2* deletion mutant. However, at 6 dpi, the infectious hyphal growth of WT strain and *UeKpp2* mutant were almost identical (Figure 6A). Relative-quantity analyses of the DNA copies of *UeActin* and *ZlActin* were used to detect the number of fungal cells to evaluate the amount of hyphae. Fewer cells were detected in the *UeKpp2* mutant at 3 dpi (Figure 6B). However, at 6 dpi, comparable amounts were observed between the mutant and WT strains (Figures 6A,B). After that, there was no obvious difference of hyphal growth status and amounts observed in the WT strain and *UeKpp2* mutant infected plants (data was not shown). Thus, even on the plant surface, the *UeKpp2* deletion only influenced infectious hyphal formation and did not impact fungal penetration or proliferation. Moreover, at 75 dpi, we observed slightly swollen stems of more than 70% both of the WT strain infected plants (31/40, swollen plants/total plants) and *UeKpp2* mutant infected plants (29/40), but no teliospores were observed in a five randomly selected plants whether infected with WT strain or *UeKpp2* mutant. At 80 dpi, we could observe teliospores in all the five randomly selected plants, no clear distinction between the plants infected with WT strain or *UeKpp2* mutant. After 90 days of culture, all of the infected plants inoculated with either the *UeKpp2* deletion mutant or the WT strains had swollen stems full of teliospores, with similar size and shape (Figures 6C,D). Additionally, the germination rate of the teliospores showed no difference between the mutant and WT strains (Figure 6E). These results indicate that UeKpp2 was not required for penetration, proliferation, teliospore formation, or germination in *U*. *esculenta*.

## Discussion

In smut fungi, the switch from yeast-like budding growth to infective filamentous growth occurs in response to environmental cues and is tightly controlled by complex genetic pathways. Among these, the cAMP-PKA and MAPK pathways are crucial to ensuring the coordination and timing of the processes (Skalhegg and Tasken, 2000; Breitkreutz et al., 2001; Martinez-Espinoza et al., 2004; Raudaskoski and Kothe, 2010). In this work, we studied the functional properties of the MAPK UeKpp2. It should be noted that the ectopic expression of *UeKpp2*, a highly conserved MAPK in smut fungi, restored defective filamentous growth and pathogenic fungal development of the *U*. *maydis kpp2* mutant SG200Δkpp2. However, in *U*. *esculenta*, UeKpp2 only participates in mating and filamentous growth *in vitro* and does not impact fungal pathogenicity. In addition, we found that a morphological change appeared in budding cells in the *UeKpp2* deletion mutant, indicating a special role for *UeKpp2* in the budding growth of *U*. *esculenta*.

### Regulation of Mating-Type Genes Through MAPK Is Weakened in *U*. *esculenta*

The MAPK and cAMP-PKA signaling pathways are necessary for pheromone responses in fungi (Maidan et al., 2005; Klosterman et al., 2007; Saito, 2010; Jung et al., 2011). In *U*. *maydis*, which is closely related to *U*. *esculenta* (Ye et al., 2017), Prf1 is a core transcription factor that regulates the expression of the *a* and *b* genes during mating (Zhang et al., 2018b). Recognition between pheromones and pheromone receptors results in the activation of MAPK cascade and PKA signaling, in which the MAPK Kpp2 further regulates Prf1 at the transcriptional level through phosphorylation, and PKA catalytic subunits are responsible for the post-transcriptional regulation of Prf1 (Kaffarnik et al., 2003). Among these, Kpp2 is not necessary for the induction of *a* genes but acts on the increased expression of the *a* genes. Additionally, Kpp2 is important for pheromone-induced expression of the *b* heterodimer (Kaffarnik et al., 2003; Muller et al., 2003; Zarnack et al., 2008; Elias-Villalobos et al., 2011). In *U*. *esculenta*, we have shown that UePrf1, a homolog of Prf1, is necessary for the regulation of the *a* and *b* genes (Zhang et al., 2018b), indicating a pheromone response mechanism that is similar to that of *U*. *maydis*. However, the expression level of *UePrf1* did not decrease during mating in *UeKpp2* mutants (Figure 4B). Moreover, the induction of the *a* and *b* genes also did not weaken (Figures 4, 5). In addition, pheromones encoded by the *mfa* genes in one haploid strain is sufficient for the conjugation tubes formation in its compatible strains (Zhang et al., 2019). So mating between UeT14ΔUeKpp2 and UeT55-EGFP induced normally functioning conjugation tubes in the UeT55-EGFP strain (Figure 4A), indicating a normal induction of *mfa* genes in *UeKpp2* mutants. Additionally, the *UeRbf1* induction strain in the *UeKpp2* mutant under the promoter of *bW2* (UeTSPΔUeKpp2:PbUeRbf1) recovered the induced expression of *UeRbf1*, the filamentous growth, and the fuzzy appearance (Figure 5G), indicating that the induction of *b* genes was not affected in *UeKpp2* mutants. These findings suggest that the impact of UeKpp2 on the regulation of mating-type genes was significantly weakened in *U*. *esculenta* compared to *U*. *maydis*. We believe that this is due to an endogenous trend in *U*. *esculenta*. Because most *U*. *esculenta* overwinter in mycelium form and reinfect directly with mycelium the next year under asexual cultivation, there is a significantly lower chance that a pheromone response will occur during the life cycle of *U*. *esculenta*. However, it should be further studied whether this is due to the endogenous life cycle of *U*. *esculenta* in *Z*. *latifolia* or there are other regulatory factors that have not yet been found.

### UeKpp2 Is Involved in the Regulation of the Morphogenesis-Related NDR Kinase Pathway in *U*. *esculenta*

In fungi, morphology and the cell cycle are intricately connected (Sartorel and Perez-Martin, 2012). Fungi impose delays or arrests at specific cell cycle stages to enable the cell to adapt to unfavorable stress conditions or to synchronize cell cycle progression before mating, which is negatively regulated by MAPK cascades (Carbó and Pérez-Martín, 2010). In response to pheromone recognition, cell cycle arrest regulated by MAPK signaling occurs in the process of budding, leading to the formation of conjugation tubes before cell fusion (Garcia-Muse et al., 2003). In yeasts, for example, cell fusion requires a previous G1 cell cycle arrest, regulated by a Fus3 MAPK cascade via phosphorylation of a cyclin-dependent kinase inhibitor Far1 (Davey, 1998). In *U*. *maydis*, the activation of Kpp2 results in a prolonged G2 phase, which is believed to result in polar extension of the cell and the formation of conjugation tubes (Garcia-Muse et al., 2003). As with *U*. *maydis*, a mutation in the pheromone response MAPK UeKpp2 significantly reduces the formation of conjugation tubes (Figures 3C, 4A).

Meanwhile, we found that the morphology of the budding cells also changed in the *UeKpp2* mutants (Figure 2A), and they became elongated and showed several buddings. This was similar to the effect of a defect in the morphogenesis-related NDR kinase (MOR) pathway that is conserved among different fungi (Maerz and Seiler, 2010). In addition, we found that a mutation of *UeUkc1*, a gene encoding the homolog to the protein kinase critical in the MOR pathway (Verde et al., 1998; Durrenberger and Kronstad, 1999), caused similar budding cell morphology to the *UeKpp2* mutants (Figure 2B). In *U*. *maydis*, bud formation takes place during the G2 phase and relies almost entirely on polar growth (Steinberg et al., 2001). Downregulation of *ukc1* results in a prolonged G2 phase and enlarged cells that are strikingly polarized (Sartorel and Perez-Martin, 2012). Thus, we further investigated the expression levels of *UeUkc1* in *UeKpp2* mutants. As expected, the expression of *UeUkc1* in the mutant fell dramatically (Figure 2D). It is worth noting that the abnormal morphology of the *UeKpp2* mutants could be restored to normal, with an increased expression of *UeUkc1*, by adding 20 mM cAMP or by inducing *UeUkc1* over-expression (Figures 2A,C,D). These results indicate that, in *U*. *esculenta*, UeKpp2 might be involved in the regulation of the MOR pathway through UeUkc1, unlike in the case of *U*. *maydis*, in which only crk1 has been proven to be responsible for the morphology of MOR mutants (Sartorel and Perez-Martin, 2012).

In addition, we found that mutant *UePkaC* cells were elongated and had several buds, with a reduced expression of *UeUkc1* (Figures 2B,D). cAMP, a signal molecule that activates the PKA pathway (Cherkasova et al., 2003), increased the expression level of *UeUkc1* and restored the cell shape to its normal form in *UeKpp2* mutants. Thus, we speculated that activating the PKA pathway could compensate for the defective cell shape when the MAPK pathway is disabled. In addition, the abnormal morphology of *UeKpp2* mutants was not restored under conditions of nitrogen starvation or stress (including cell wall stress, hyperosmotic stress, and oxidative stress). However, an almost normal budding of the two comparable *UeKpp2* mutants was observed during mating (Figure 3C). These phenomena led us to suspect that pheromone signaling may activate the PKA pathway to compensate for the defective cell shape when the MAPK pathway is disabled, where environmental cues do not. However, all of these speculations require further evidence.

### Role of UeKpp2 in Induction of *UeRbf1* in Filamentous Growth Regulation Is Only Observed *in vitro*

In *U*. *maydis*, *b*-dependently induced *Rbf1* is required for *b*-dependent filament formation and sufficient for filament formation in the absence of an active bE/bW heterodimer (Heimel et al., 2010a). Further, Rbf1 may be regulated by Prf1 through an unknown mechanism (Heimel et al., 2010a). In *U*. *esculenta*, the *b* genes also play an important role in the growth of the dikaryotic filament during mating *in vitro* and *in vivo* (Zhang et al., 2019). However, in the *UeKpp2* mutant with the UeTSP strain background, which can spontaneously form filaments *in vitro* and *in vivo* (Zhang et al., 2019) and avoid the influence of conjugation tube formation, filamentous growth was also seriously affected (Figure 5F). During these developmental steps, the expression of the *b* genes in the mutants was similar to that in the WT strains, while *UeRbf1* expression decreased significantly in mutants (Figures 5E,G). It was believed that the defective filamentous growth in *UeKpp2* mutants was related to a defect in the induction of *UeRbf1*, not in the induction of *b* genes. As with *U*. *maydis* (Heimel et al., 2010a), the deletion of *UeRbf1* in UeTSP strains could not form infectious hyphae (Supplementary Figure S6). However, this was different from *U*. *maydis* in that *UeKpp2* deletion mutants with the UeTSP strain did not affect the growth of infectious hyphae in the host at all, and the mutation of UeKpp2 did not affect the induction of galls or the formation or germination rate of teliospores (Figure 6 and Supplementary Figure S6). Hence, in *U. esculenta*, the role of UeKpp2 in the induction of *UeRbf1* independently from bE/bW heterodimer only happened *in vitro* during mating in our study. This may be related to the endogenous life cycle of *U*. *esculenta* in *Z*. *latifolia*, such that the mating process with comparable haploid strains rarely appeared. However, further discussion of how UeKpp2 participates in the distinct developmental stages of the life cycle of *U*. *esculenta* in response to different external signals is necessary.

## Data Availability Statement

All datasets generated for this study are included in the article/Supplementary Material.

## Author Contributions

YZ and ZY conceived and designed the experiments, and wrote and revised the manuscript. YH, QC, YY, and YZ performed the experiments. YH, QC, WX, HC, and XY analyzed the data. YZ and YH prepared the figures and tables. All authors have read and approved the final manuscript.

## Conflict of Interest

The authors declare that the research was conducted in the absence of any commercial or financial relationships that could be construed as a potential conflict of interest.

## Supplementary Material

FIGURE S1 Identification of the derived strains SG200Δkpp2::Kpp2-3 and SG200Δkpp2::UeKpp2-6. **(A)** The single-copy case was verified by Southern blot analyses. “M” stands for HindIII marker. “1” strands for the test strain SG200Δkpp2::Kpp2-3. “2” strands for the test strain SG200Δkpp2::UeKpp2-6. **(B)** The expression level was analyzed by qRT-PCR. ** denotes significant difference at *p* < 0.01.

FIGURE S2 Identification of the derived mutants. **(A–C)** Southern blot analyses. “M” stands for HindIII marker. **(A)** “1” strands for the test strain UeT14ΔUeKpp2. “2” strands for the test strain UeT55ΔUeKpp2. “3” strands for the test strain UeTSPΔUeKpp2. **(B)** “2” strands for the test strain UeT55ΔUePkaC. “3” strands for the test strain UeT14ΔUeUkc1. “4” strands for the test strain UeT55ΔUeUkc1. “5” strands for the test strain UeT14ΔUePkaC. **(C)** “1” strands for the test strain UeTSPΔUeKpp2::PbUeRbf1. “2” strands for the test strain UeTSPΔUeRbf1. **(D–G)** PCR verification of UePrf1 deletion mutants. “M” stands for DL2000 marker. The fragments that are verified from top to bottom are target gene, hygromycin resistance gene, upstream segment of target gene and downstream segment of target gene. **(D)** “1” strands for the test strain UeT14. “2” strands for the test strain UeT14ΔUeKpp2. “3” strands for the test strain UeT55ΔUeKpp2. “4” strands for the test strain UeTSPΔUeKpp2. “5” strands for the test strain UeT55. **(E)** “1” strands for the test strain UeT14ΔUePkaC. “2” strands for the test strain UeT55?UePkaC. “3” strands for the test strain UeT14. “4” strands for the test strain UeT55. **(F)** “1” strands for the test strain UeT14ΔUeUkc1. “2” strands for the test strain UeT55ΔUeUkc1. “3” strands for the test strain UeT14. “4” strands for the test strain UeT55. **(G)** “1” strands for the test strain UeTSP. “2” strands for the test strain UeTSPΔUeRbf1. “3” strands for the test strain UeTSPΔUeKpp2::PbUeRbf1. **(H–K)** The expression level of target genes was analyzed by qRT-PCR. ** denotes significant difference at *p* < 0.01.

FIGURE S3 Assessment of tolerance of WT or *UeKpp2* mutant toward stressful conditions. **(A)** Serially diluted cells (from about 10^7^ to 10^4^ / mL) of WT or *UeKpp2* mutant were spotted onto YEPS medium, YEPS supplemented with H_2_O_2_ (1 mM), Congo Red (0.5 mM), or NaCl (500 mM) and BM medium separately. Images were separately taken 3 days post spotting.

FIGURE S4 qPCR verification of the expression level of *UeKpp2* **(A)** or *UeUkc1* **(B)** expressed through the constitutive promoter in *UeKpp2* mutants. *Denotes significant difference at *p* < 0.05.

FIGURE S5 Amino acid sequences arrangement. **(A)** Amino acid sequences in the arrangement of UeUkc1 protein (MN845072) and its fungal orthologs: *U. maydis* Ukc1 (AAC09291.1), *U. hordei* Ukc1(CCF50109.1), *Neurospora crassa* Cot1 (XP_962150.2), *Schizosaccharamyces Pombe* Orb6 (NP_593165.1). The black and gray shadows denote identical and conserved residues, respectively. The underline represents STKC-NDR-like-fungal domains. **(B)** Arrangement of amino acid sequences of UePkaC protein (ALM02104.1) and its fungal orthologs: *U. maydis* PkaC (XP_011391132.1), *Neurospora crassa* PkaC (ACA48490.1), *Colletotrichum trifolii* PkaC (AAC04355.1). The black and gray shadows denote identical and conserved residues, respectively. Underlining represents STKc-PKA-like domains.

FIGURE S6 Fungal infection status in leaf sheath. Seedlings infected with the UeTSP strain, the corresponding *UeKpp2* deletion strain, and the *UeRbf1* deletion strain were collected at 3 and 6 dpi, stained with wheat germ agglutinin-Alexa Fluor 488 and analyzed by laser scanning confocal microscopy. The scale bar represents 50 μm.

TABLE S1 Strains used in this study.

TABLE S2 Primers used in this study.
