# Supplementary figures and images for: Functional Properties of the MAP Kinase UeKpp2 in Ustilago esculenta
Source: Front Microbiol. 2020 Jun 9;11:1053. doi: 10.3389/fmicb.2020.01053 (PMC7295950; doi:10.3389/fmicb.2020.01053)

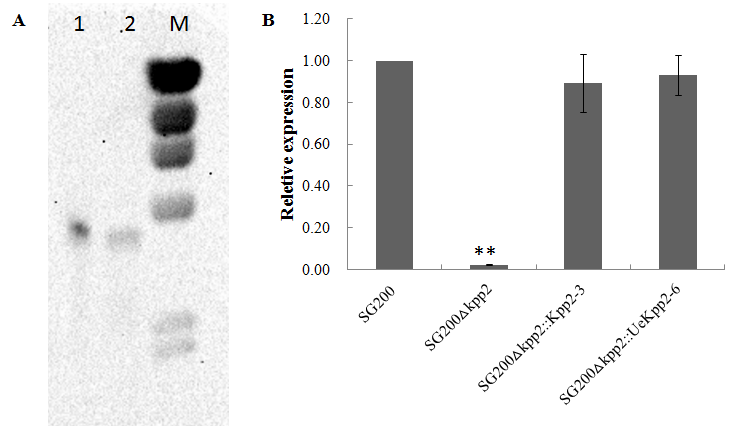

Supplement: FIGURE S1 — Identification of the derived strains SG200Δkpp2::Kpp2-3 and SG200Δkpp2::UeKpp2-6. (A) The single-copy case was verified by Southern blot analyses. “M” stands for HindIII marker. “1” strands for the test strain SG200Δkpp2::Kpp2-3. “2” strands for the test strain SG200Δkpp2::UeKpp2-6. (B) The expression level was analyzed by qRT-PCR. ** denotes significant difference at p < 0.01. [file Image_1.TIF]

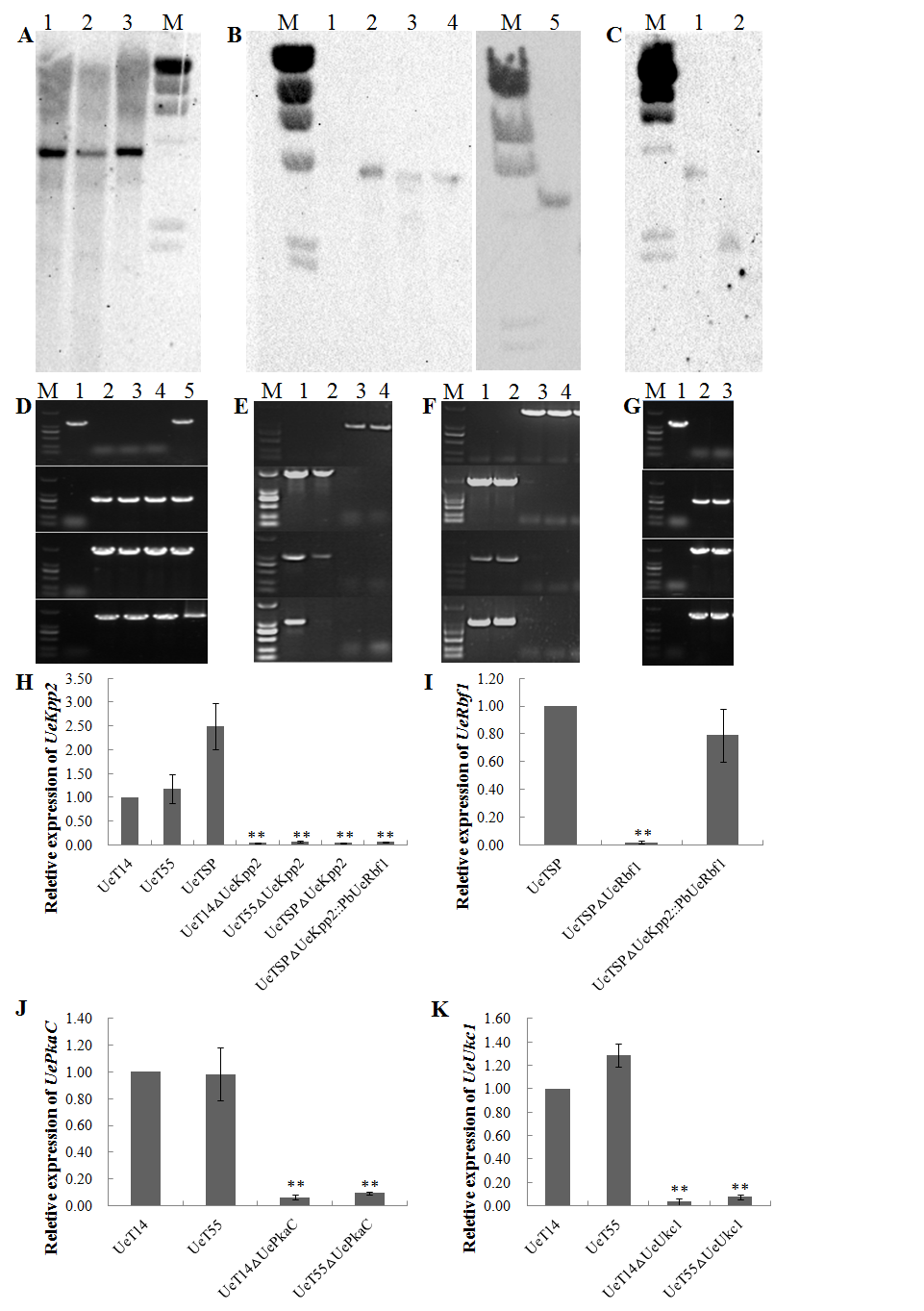

Supplement: FIGURE S2 — Identification of the derived mutants. (A–C) Southern blot analyses. “M” stands for HindIII marker. (A) “1” strands for the test strain UeT14ΔUeKpp2. “2” strands for the test strain UeT55ΔUeKpp2. “3” strands for the test strain UeTSPΔUeKpp2. (B) “2” strands for the test strain UeT55ΔUePkaC. “3” strands for the test strain UeT14ΔUeUkc1. “4” strands for the test strain UeT55ΔUeUkc1. “5” strands for the test strain UeT14ΔUePkaC. (C) “1” strands for the test strain UeTSPΔUeKpp2::PbUeRbf1. “2” strands for the test strain UeTSPΔUeRbf1. (D–G) PCR verification of UePrf1 deletion mutants. “M” stands for DL2000 marker. The fragments that are verified from top to bottom are target gene, hygromycin resistance gene, upstream segment of target gene and downstream segment of target gene. (D) “1” strands for the test strain UeT14. “2” strands for the test strain UeT14ΔUeKpp2. “3” strands for the test strain UeT55ΔUeKpp2. “4” strands for the test strain UeTSPΔUeKpp2. “5” strands for the test strain UeT55. (E) “1” strands for the test strain UeT14ΔUePkaC. “2” strands for the test strain UeT55?UePkaC. “3” strands for the test strain UeT14. “4” strands for the test strain UeT55. (F) “1” strands for the test strain UeT14ΔUeUkc1. “2” strands for the test strain UeT55ΔUeUkc1. “3” strands for the test strain UeT14. “4” strands for the test strain UeT55. (G) “1” strands for the test strain UeTSP. “2” strands for the test strain UeTSPΔUeRbf1. “3” strands for the test strain UeTSPΔUeKpp2::PbUeRbf1. (H–K) The expression level of target genes was analyzed by qRT-PCR. ** denotes significant difference at p < 0.01. [file Image_2.TIF]

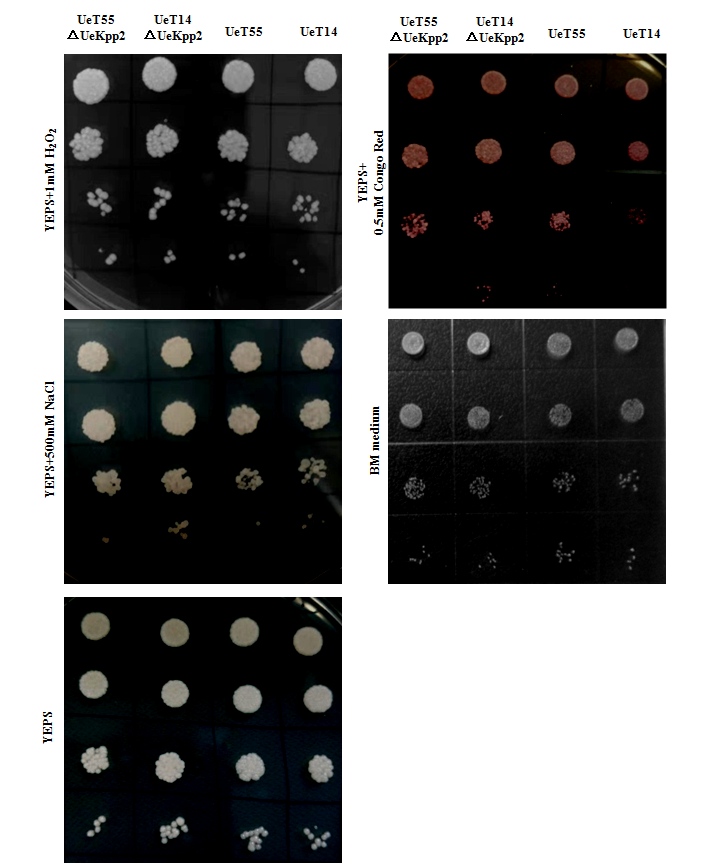

Supplement: FIGURE S3 — Assessment of tolerance of WT or UeKpp2 mutant toward stressful conditions. (A) Serially diluted cells (from about 107 to 104 / mL) of WT or UeKpp2 mutant were spotted onto YEPS medium, YEPS supplemented with H2O2 (1 mM), Congo Red (0.5 mM), or NaCl (500 mM) and BM medium separately. Images were separately taken 3 days post spotting. [file Image_3.TIF]

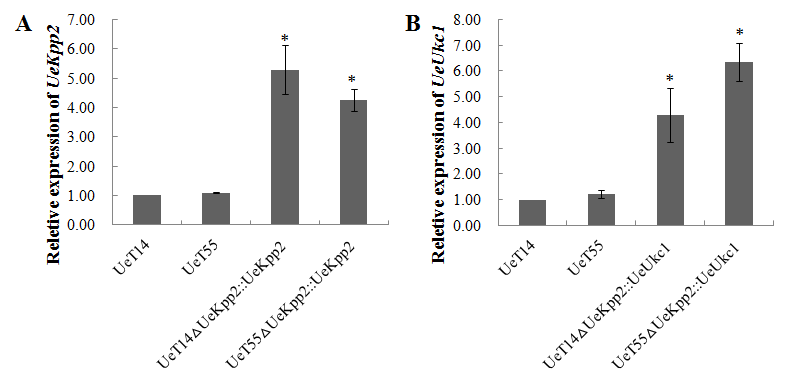

Supplement: FIGURE S4 — qPCR verification of the expression level of UeKpp2 (A) or UeUkc1 (B) expressed through the constitutive promoter in UeKpp2 mutants. *Denotes significant difference at p < 0.05. [file Image_4.TIF]

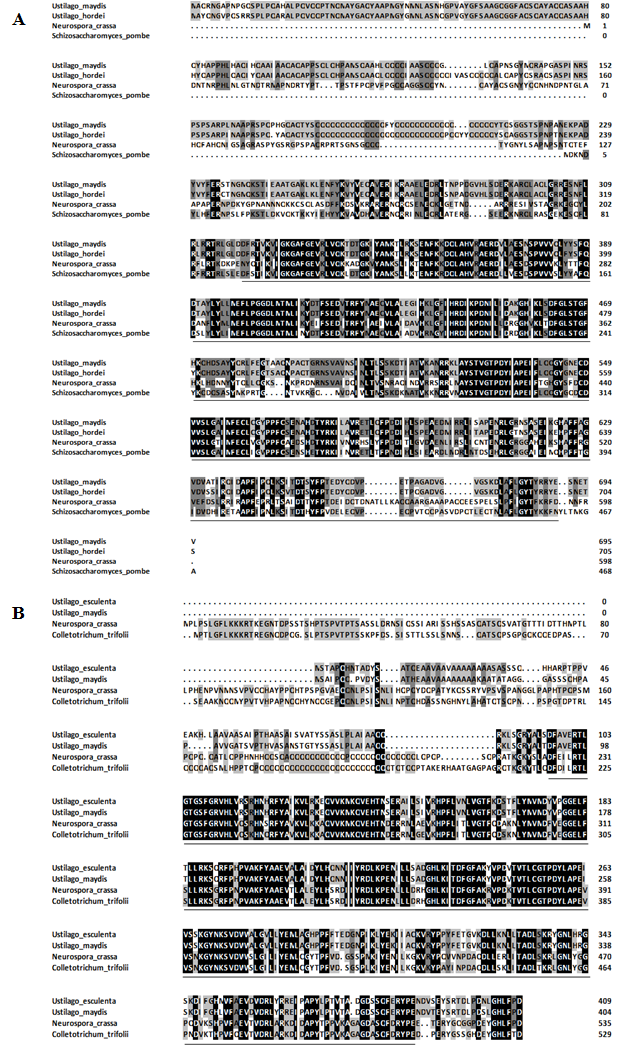

Supplement: FIGURE S5 — Amino acid sequences arrangement. (A) Amino acid sequences in the arrangement of UeUkc1 protein (MN845072) and its fungal orthologs: U. maydis Ukc1 (AAC09291.1), U. hordei Ukc1(CCF50109.1), Neurospora crassa Cot1 (XP_962150.2), Schizosaccharamyces Pombe Orb6 (NP_593165.1). The black and gray shadows denote identical and conserved residues, respectively. The underline represents STKC-NDR-like-fungal domains. (B) Arrangement of amino acid sequences of UePkaC protein (ALM02104.1) and its fungal orthologs: U. maydis PkaC (XP_011391132.1), Neurospora crassa PkaC (ACA48490.1), Colletotrichum trifolii PkaC (AAC04355.1). The black and gray shadows denote identical and conserved residues, respectively. Underlining represents STKc-PKA-like domains. [file Image_5.TIF]

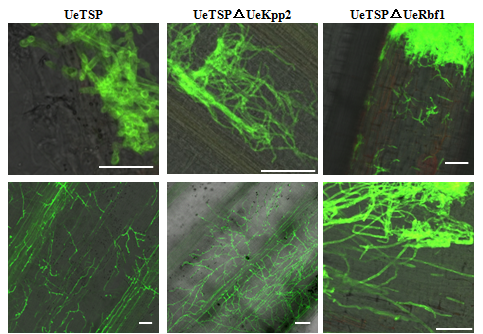

Supplement: FIGURE S6 — Fungal infection status in leaf sheath. Seedlings infected with the UeTSP strain, the corresponding UeKpp2 deletion strain, and the UeRbf1 deletion strain were collected at 3 and 6 dpi, stained with wheat germ agglutinin-Alexa Fluor 488 and analyzed by laser scanning confocal microscopy. The scale bar represents 50 μm. [file Image_6.TIF]
