# Supplementary material for: Functional Properties of the MAP Kinase UeKpp2 in Ustilago esculenta
Source: Front Microbiol. 2020 Jun 9;11:1053. doi: 10.3389/fmicb.2020.01053 (PMC7295950; doi:10.3389/fmicb.2020.01053)
Supplement: TABLE S1 — Strains used in this study. [file Table_1.docx]

**Table S1 Strains used in this study.**

| **Brief description** | **Resistence** | **Reference** |
| --- | --- | --- |
| Wild type strain with *a1b1* genotype | *--* | Zhang et al., 2019 |
| Wild type strain with *a2b2* genotype | *--* | Zhang et al., 2019 |
| *EGFP* over-expression in UeT55 |  |  |
| *UeKpp2* deletion in UeT14 | Hygromycin B | This study |
| *UeKpp2* deletion in UeT55 | Hygromycin B | This study |
| *UeKpp2* overexpression in *UeKpp2* deletion strain UeT14△UeKpp2 | Hygromycin B and carboxin | This study |
| *UeKpp2* overexpression in *UeKpp2* deletion strain UeT55△UeKpp2 | Hygromycin B and carboxin | This study |
| *UePkaC* deletion in UeT14 | Hygromycin B | This study |
| *UePkaC* deletion in UeT55 | Hygromycin B | This study |
| *UeUkc1* deletion in UeT14 | Hygromycin B | This study |
| *UeUkc1* deletion in UeT55 | Hygromycin B | This study |
| *UeUkc1* overexpression in *UeKpp2* deletion strain UeT14△UeKpp2 | Hygromycin B and carboxin | This study |
| *UeUkc1* overexpression in *UeKpp2* deletion strain UeT55△UeKpp2 | Hygromycin B and carboxin | This study |
| *mfa1.2* and *bE1* expressed with their own promoter in UeT55 | Carboxin | Zhang et al., 2019 |
| *UeKpp2* deletion in UeTSP | Hygromycin B and carboxin | This study |
| *UeRbf1* deletion in UeTSP | Hygromycin B and carboxin | This study |
| *UeRbf1* expressed with *bW2* promoter in *UeKpp2* deletion strain UeTSP△UeKpp2 | Hygromycin B and carboxin | This study |
| *solopathogen haploid strain with a1mfa2 b1bW2 genotype* | *--* | Bölker et al., 2011 |
| *Kpp2* deletion in SG200 | Hygromycin B | Müller et al., 2000 |
| *Kpp2* complementary strain*; Kpp2* expressed with its own promoter in *Kpp2* deletion strain SG200△kpp2 | Hygromycin B and carboxin | This study |
| *Kpp2* complementary strain*; UeKpp2* expressed with thepromoter of *Kpp2* in *Kpp2* deletion strain SG200△kpp2 | Hygromycin B and carboxin | This study |
