# Supplementary material for: Functional Properties of the MAP Kinase UeKpp2 in Ustilago esculenta
Source: Front Microbiol. 2020 Jun 9;11:1053. doi: 10.3389/fmicb.2020.01053 (PMC7295950; doi:10.3389/fmicb.2020.01053)
Supplement: TABLE S2 — Primers used in this study. [file Table_2.docx]

**Table S2 Primers used in this study.**

| Primer | Sequence (5’→3') | purpose |
| --- | --- | --- |
| Uekpp2-UF | GACCTGGTTCGTAAAGCTGT | genes deletion mutants construction |
| Uekpp2-UR | GTAGTTACCACGTTCGGCCATTTGTTCGTACAATGCCAAAC |  |
| Uekpp2-DF | TGTCAAACATGAGGCCTGAGTCGGGGAAGTAAAAGAAGGG |  |
| Uekpp2-DR  UePkaC-UF | GTGGATCATGACCGATGAGC  AGGAACTTGCCCGACAAGCTT |  |
| UePkaC -UR | ACATGAGGCCTGAGTGTCTACAGAATGGATGGAGC |  |
| UePkaC -DF | TTTCGGCCATCTAGGCTCGCCCCCTCTCCTTTCTTC |  |
| UePkaC -DR | ACCTCTCTTCCGCATCTCAT |  |
| UeRbf1-UF | TGAATTCGAGCTCGGTACCCTTAGGTCGGTTGACCAAAGC |  |
| UeRbf1 -UR | TCTAGAGGATCCCCGGTACCCCTGTGTTGACACGAGTTTC |  |
| UeRbf1 -DF | ACCTGCAGGCATGCAAGCTTCGTCTCTAGTCCGCTTCAAC |  |
| UeRbf1 -DR | ACCATGATTACGCCAAGCTTTAAGTACAACGCTGTCATCC |  |
| UeUkc1-UF | GTGAATTCGAGCTCGGTACCGAGCAACAAGACGGTCTCTG |  |
| UeUkc1 -UR | CTAGAGGATCCCCGGGTACCAAACGCGTGGGTGCGAATAG |  |
| UeUkc1 -DF | GACCTGCAGGCATGCAAGCTTCAATGGTAGATGCGCGTTGG |  |
| UeUkc1 -DR  Hyg3 | ACCATGATTACGCCAAGCTTCGGATACCACCTCCAAGACC  GGATGCCTCCGCTCGAAGTA |  |
| Hyg4 | CGTTGCAAGACCTGCCTGAA |  |
| HygF | TGGCCGAACGTGGTAACTAC |  |
| HygR | CTCAGGCCTCATGTTTGACA |  |
| bW2-PF | GATTGCTGTATCGGCAGGGA | plasmid construction for generation of strain UeTSP△UeKpp2::P_b_UeRbf1 |
| bW2-PR | GGTGTCTGGCAGCTTTCTCG |  |
| UeRbf1-CF | ATGGACATCCTTGGTGAGTAT |  |
| UeRbf1-CR  Uekpp2-DF1 | TCATGACGAGGAAGCGACTG  CAGTCGCTTCCTCGTCATGA GACCTGGTTCGTAAAGCTGT |  |
| Uekpp2-verity-F | ATGGCGCACGCACATGGAC | strains verification |
| Uekpp2-verity-R | TCAACGCATGATCTCATTGTAG |  |
| UePkaC -verity-F | CAGCCAGCATTTCGTACAGC |  |
| UePkaC -verity-R | CAAGTTGAGTGGCAGGTATG |  |
| UeRbf1-verity-F | GAGTTCGTCCTATCCAGATG |  |
| UeRbf1-verity-R | AGCTGCATGCTGCTGGAGAG |  |
| UeUkc1-verity-F | CTCGCTCTTTGCGTGACAAC |  |
| UeUkc1-verity-R | ACAGCCTGCTCCACGTACAC |  |
| Hyg-verity-F | TAAGCTGCCGAGTAACGTCAC |  |
| Hyg-verity-R | CATCGCAAGACCGGCAACAG |  |
| MF167 | AACTCGCTGGTAGTTACCAC |  |
| MF168 | ACTAGATCCGATGATAAGCTG |  |
| Cbx-verity-F | ATGTCGCTATTCAACGTCAG |  |
| Cbx -verity-R | TTACGACGAAGCCATGATAG |  |
| kpp2-verity-F | GCTGCTTCGTCACTTTAACC |  |
| kpp2-verity-R | CCAAGGTGAGCGAGAGTTGG |  |
| Uekpp2-F3 | GCTTTGAACCGTTTGTGAGC |  |
| Uekpp2-R3 | TATACAGCGAAGTCGCCAAC |  |
| UePkaC -F3 | TCACGTCCTTTCGCGGCTTC |  |
| UePkaC -R3 | GTACTCGTACTCGCTCTCGG |  |
| UeRbf1-F3 | CAGCGCCGCATTGTTATCAG |  |
| UeRbf1-R3 | TGAGGAAACCTCCTGACTCG |  |
| UeUkc1-F3 | GTTGGCATGCGAACGCGAAC |  |
| UeUkc1-R3 | GCCAAGGAGGTGATTCTGTG |  |
| Uekpp2-qPCR-F | CACCTTGGAAATCCTGGGCA | qRT-PCR |
| Uekpp2-qPCR-R | GACGGCGAGAGGATTAGCGTT |  |
| UePkaC -qPCR-F | ACGCAAGTTGAGTGGCAGGTA |  |
| UePkaC -qPCR-R | AGAACGAACGAGGTGGACGC |  |
| UeRbf1-qPCR-F | GACCAAGCATACCCGTCGCA |  |
| UeRbf1-qPCR-R | GGAAGGTGGAGCGGTTGTGA |  |
| UeUkc1-qPCR-F | CGCGGTCAACTCGATCAAC |  |
| UeUkc1-qPCR-R | CTTGCTGCAGGAAGATCTC |  |
| mfa1.2-QF | TTCCATCTTCACTCAGCACGC |  |
| mfa1.2-QR | AGGCGACAATACATGTGGAG |  |
| pra1-QF | TCCAACCTTGTCATCGCACGAA |  |
| pra1-QR | CGATATGAGTAGATCGATGATG |  |
| mfa2.1-QF | GTTCACTATCTTCGAGACTGTTGC |  |
| mfa2.1-QR | TAGGCCACAACGCAGTAGTTG |  |
| pra2-QF | GTCTTCTCAACATTCAGGCCTGTCT |  |
| pra2-QR | TGAGATAAAATTGTGCAACCGAG |  |
| bE1-QF | AGAGCCCTGACATTCTTTCC |  |
| bE1-QR | GTGCTTCCGAGACCACAGT |  |
| bE2-QF | AGGACACCACCGACCAGAT |  |
| bE2-QR | TGAGAACAACAGCCGCTTC |  |
| bW1-QF | TCTTGACCGCTTGTCCATC |  |
| bW1-QR | GTCGTCCTAGTTCTTGCTCGT |  |
| bW2-QF | ATGTCCAAACAGCAACCAGC |  |
| bW2-QR | CGAAAAGGCGGAGAAGTGAT |  |
| UePrf1-qPCR-F | GAAGCGTTATTGAGCCTGTCG |  |
| UePrf1-qPCR-R | TTCTGGGATTGGCACTCTTGTC |  |
| β-Actin-QF | CAATGGTTCGGGAATGTGC |  |
| β-Actin-QR | GGGATACTTGAGCGTGAGGA |  |
| ZlActin-QF  ZlActin-QR | GACGGTGAGGATATCAAGCC  GCGAGGGCAACCGACAATAC |  |
| PF1 | TATAGAACTCGAGCAGCTGAGCGTCACGTTAGGCAGTCAG | complementation of *Ue**Kpp2*/*Kpp2* deletion strain and construction of *UeKpp2/UeUkc1* overexpression strains in *UeKpp2* mutants |
| PR1 | GTCCATGTGCGTGCGCCATCTTGGTTGGGTCTTTCTAGC |  |
| PR2 | TGTCCGTGGGCATGTGACATCTTGGTTGGGTCTTTCTAGC |  |
| Kpp2-CF | ATGTCACATGCCCACGGACA |  |
| Kpp2-CR | CTCACCATACCAGGACCAGGACGCATGATCTCGTTATAAA |  |
| UeKpp2-CF | ATGGCGCACGCACATGGAC |  |
| UeKpp2-CR | CTCACCATACCAGGACCAGGACGCATGATCTCATTGTAG |  |
| UeKpp2-CF1 | TATAGAACTCGAGCAGCTGAATGGCGCACGCACATGGAC |  |
| UeUkc1-CF1 | TATAGAACTCGAGCAGCTGAATGACTTACCGTAACGGCGC |  |
| UeUkc1-CR | CTCACCATACCAGGACCAGGCAGAACGGTTTCGTTACTCT |  |
